# Herpesviruses show consistency in their nuclear egress-associated induction of lamina-depleted areas as measured in an autofluorescent reporter cell model

**DOI:** 10.1186/s12985-026-03334-8

**Published:** 2026-10-20

**Authors:** Kishore Dhotre, Lena Weghorn, Martin Schütz, Manfred Marschall

**Affiliations:** 1 Harald zur Hausen Institute of Virology, Friedrich-Alexander University of Erlangen- Nürnberg (FAU), Erlangen, Germany; 2 La Jolla Institute for Immunology, San Diego, CA USA

**Keywords:** Human pathogenic herpesviruses, Viral nuclear egress, Egress-mediated rearrangement of the nuclear lamina, Virus-induced formation of lamina-depleted areas (LDAs), LDA quantitation in a reporter cell model, Consistency in LDA-specific phenotypes of α-/β-/γ-herpesviruses

## Abstract

Human herpesvirus infections span a wide variety of pathogenic outcomes, with individual representatives comprising a huge clinical relevance on human health. Translational research efforts are directed towards the improvement of broadly applicable preventive measures and detailed understanding of viral pathogenesis. Here, we studied rate-limiting steps in viral replication that show high mechanistic conservation among the *Herpesviridae* family members, in particular concerning the viral nucleocytoplasmic capsid egress. As characterized for several herpesviruses, viral nuclear egress is facilitated by the virus-induced local nuclear lamina rearrangements of infected cells. Hereby, the virus-mediated site-specific phosphorylation at lamin A/C Ser22, followed by *cis/trans* isomerization of the structural conformation, leads to the induction of lamina-depleted areas (LDAs). We have generated an LDA reporter system in primary human fibroblasts (HFFs), which conditionally express the autofluorescent lamin A/C-RFP fusion construct, for quantifying viral nuclear egress activity. This system was successfully applied for human and animal cytomegaloviruses as well as a selection of α-, β-, and γ-herpesviruses shown here. Our results illustrate that (i) the reporter system was effective in a quantitative way for all viruses analyzed; (ii) the process of LDA formation showed consistency among herpesviruses; (iii) the impairment of viral kinase activity, responsible for lamin A/C Ser22 phosphorylation, significantly reduced LDA formation; and (iv) inhibitory small molecules, directed either to the viral nuclear egress complex or the cellular isomerase activity, likewise provide an LDA-blocking effect leading to strong antiviral efficacy. Thus, the autofluorescent reporter cell model provides manifold options to study herpesviral productive replication, nuclear host interaction, and the pathogenesis-related impact of regulatory factors.

## Introduction

The nuclear lamina is a meshwork of intermediate filaments that maintains the shape of the nucleus by providing mechanical stability and harbors additional interactive and regulatory functions [1, 2]. The nuclear lamins of type A are expressed from the human LMNA gene, which includes alternative splicing, thus giving rise to the A and C proteins, termed as lamin A/C that share an identical N-terminal portion. During the process of cellular mitosis, the tightly assembled nuclear lamina is reversed by disassembly of the head-to-tail-intertwined lamin dimers and polymers, which normally takes place in the early prophase to reassemble later during telophase. Interestingly, the mechanism of nuclear lamina disassembly is exploited by herpesviruses to promote their nucleocytoplasmic egress of capsids [3–6]. In this case, the fine-regulated process of herpesvirus-induced disassembly is confined to locally restricted spots of the nuclear envelope, described as lamina-depleted areas (LDAs) [7–9]. The family *Herpesviridae* is composed of medically relevant human viruses, which are widely different in their pathogenicity, but all characterized by the establishment of a lifelong latency with recurrent reactivations [10]. The large virions (up to 200 nm) carry a double-stranded DNA genome and acquire host membranes to mature into enveloped particles [11, 12]. The three subfamilies are based on the prototype species of herpes simplex virus type 1 (HSV-1, α-herpesviruses), human cytomegalovirus (HCMV, β-herpesviruses), and Epstein-Barr virus (EBV, γ-herpesviruses).

Due to the large size of the herpesviral nuclear capsids (~125 nm in diameter), the nuclear pore complex (NPC, ~39 nm) does not permit nucleocytoplasmic transport [13]. To this end, herpesviruses have evolved an NPC-independent mechanism of nuclear envelope (NE) transition, which is based on the formation of a viral core nuclear egress complex (NEC) [3, 13–23]. Thus, the rate-limiting step of nuclear capsid egress is regulated by the viral core NEC, which is formed as a heterodimer between the inner nuclear membrane (INM)-associated groove protein (pUL50 in the case of HCMV) and its nucleoplasmic interaction partner carrying a hook-like N-terminal binding domain (pUL53 in the case of HCMV). The structural details of these viral NEC proteins have been resolved recently, and the comparison of 3D-structures between α-, β-, and γ-herpesviral NECs suggested a high degree of conservation [24, 25]. This core NEC, on the one hand, carries the capacity to oligomerize into hexameric assemblies and lattice-like patterns [16], and, on the other hand, recruits several NEC-associated factors, such as the viral ortholog of cyclin-dependent kinases (CDK), i.e. pUL97 in the case of HCMV [26], bridging factors such as the p32/gC1qR (p32) [27], and the host peptidyl-prolyl cis/trans isomerase Pin1 [28].

Through the concerted action of NEC-associated regulatory factors, lamin A/C undergoes site-specific phosphorylation at serine 22 (pSer22), as mediated by HCMV pUL97 [7] or related herpesviral kinases (i.e. HSV-1 pUS3/pUL13 [29, 30], VZV ORF66/ORF47 [31], HHV-6 pU69 [32], EBV BGLF4 [33], and others). This phospho-specific triggering activates factors of the multicomponent NEC to proceed in the nuclear lamina rearrangement. Hereby, the nuclear lamina is locally destabilized and rearranged in the form of LDA induction. Then, capsids traverse the NE through the inner nuclear membrane (INM) into the perinuclear space and subsequently undergo de-envelopment at the outer nuclear membrane (ONM) to exit into the cytoplasm [34, 35]. Especially, LDA formation is dependent on the activity of Pin1, which is a multidomain protein consisting of the WW binding domain and the catalytic isomerase domain, recognizes lamin A/C pSer22 in order to catalyze its N-terminal *cis/trans* isomerization [7]. This regulatory step disintegrates the polymeric lamin A/C structure, particularly leading to LDA formation, which represents a coherently shared property of herpesviruses [9, 14, 36]. Several studies have consistently emphasized the central importance of local disassembly of nuclear lamina to drive the efficiency of herpesviral nuclear capsid egress [7, 9, 36, 37].

Recently, we reported the coherence of quantifiable phenotypic changes in the cellular NE by virus LDA formation, as exemplified for the human, chimpanzee, and rhesus macaque CMV infections (HCMV, ChCMV, and RhCMV) of primary human fibroblasts (HFFs). The recombinant HFF populations, expressing lamin A/C-RFP in a doxycycline-inducible manner, have been used as a cellular model to study the viral nuclear egress mechanism [9]. In this study, we extended the model to investigate LDA formation in a comparative setting, using representative herpesviruses of the three subfamilies, i.e. HCMV, HSV-1, VZV, EBV, and MHV-68. Site-specific pSer22 phosphorylation, LDA formation, and significant reduction of LDAs under treatment with NEC-inhibitory small molecules were concordantly detected. Hence, our novel data underline the usefulness of this cellular reporter system for the study of herpesvirus infections and underlines the conserved mode of nuclear egress within herpesvirus subfamilies. Notably, the specific application of this model for the discovery of NEC-directed, broadly acting antiviral prototype drugs is discussed.

## Methods

### Cell culture and viruses

The primary human foreskin fibroblasts (HFFs) were maintained in minimal essential medium (MEM, 21090022; Thermo Fisher Scientific, Waltham, MA, USA), and supplemented with 10% fetal bovine serum (FBS, F7524; Sigma Aldrich, Steinheim, Germany), 1x GlutaMAX^TM^ (35050038; Thermo Fisher Scientific, Waltham, MA, USA), and 10 μg/ml gentamicin and were incubated at standard incubation conditions of 37 °C, 5% CO_2_, and 80% humidity. The selected recombinant HFF cell populations expressing lamin A/C tagged with RFP were alternatively supplemented with tetracycline-negative FBS (FBS-TET-12A; Capricorn Scientific, Ebsdorfergrund, Germany) and 500 µg/ml geneticin (G418, 10131035; Thermo Fisher Scientific, Waltham, MA, USA) as a positive selection marker and were induced for recombinant lamin A/C-RFP by refreshing 500 ng/ml doxycycline every third day. SVK-CR2 and Vero 76 cells were cultivated in Dulbecco’s modified Eagle’s medium (DMEM) (Thermo Fisher Scientific, Waltham, MA, USA) and supplemented with 10% FBS, 1× GlutaMAX™, and 10 µg/ml gentamicin. The following virus stocks were used in this study: HCMV AD169, AD169-GFP, AD169-GFP-based viral kinase mutants ΔORF-UL97 and UL97 Δ231-280, MHV-68-GFP, MHV-68-Luc, EBV Akata-BX1-GFP, VZV Oka-GFP, and HSV-1 VP26-GFP [8, 27, 38–43].

### Antibodies

The primary antibodies used for the present study were as follows: mouse mAb-β-actin AC-15 (Sigma-Aldrich, Steinheim, Germany), rabbit mAb-Lamin A/C (EPR4100; Abcam plc), rabbit polyclonal antibody pAb-pSer22 Lamin A/C (Cell Signaling Technology), mouse mAb-Pin1 (SC46660; Santa Cruz Biotechnology), mouse mAb-IE1 recognizing HCMV immediate early protein IE1p72 (kindly provided by William Britt, Univ. Birmingham, AL, USA), GFP; mouse mAb-GFP (7.1 and 13.1; Roche Life Science), mAb-UL97.01 (kindly provided by T. Lenac and S. Jonick; Univ. Rijeka, Croatia), mAb-UL50.01 (kindly provided by S. Jonjic and T. Lenac Rovis), mAb-UL53.01 (kindly provided by Prof. Stipan Jonjic and Dr. Tihana Lenac Rovis, University of Rijeka, Rijeka, Croatia), mAb pp65 (HCMV, 65-33, kindly provided by Prof. William Britt, University Birmingham, AL, USA), mAb-ICP0 recognizing HSV-1 immediate early protein ICP0 (SC53070; Santa Cruz Biotechnology). As secondary antibodies, we used HRP-conjugated anti-mouse/- rabbit antibodies (for Wb analyses; Jackson ImmunoResearch) and Alexa Fluor™ 488-/647-conjugated antibodies (for IF analyses; A-11008, Thermo Fisher Scientific).

### Western blot (Wb) analysis

Wb analysis of proteins of interest was carried out in accordance with standard protocol described elsewhere [44, 45].

### Confocal imaging for the quantitation of nuclear lamina deformation in herpesvirus-infected cells

In order to visualize the herpesvirus-induced deformation of the nuclear lamina, the recombinant HFFs expressing lamin A/C-RFP were seeded in a 6-well plate at a seeding density of 3x10^5^ cells/well on a coverslip. In order to express the recombinant lamin A/C-RFP, cells were induced with doxycycline (dox, 500 ng/ml) under repeated refreshment every third day. Further, 2 d post-seeding, the dox-induced recombinant lamin A/C-RFP HFFs were infected with HSV-1 VP26-GFP (MOI 1), VZV Oka-GFP (pre-infected HFFs at a seeding density of 25% of the total HFFs), HCMV AD169-GFP (MOI 2), and MHV-68-GFP (MOI approx. 0.25). EBV-permissive SVK-CR2 epithelial cells were seeded and infected with EBV Akata-BX1-GFP stock virus (MOI approx. 0.25; produced with Akata-BX1 cells, having been chemically induced by 40 ng/ml of 12-O-tetradecanoylphorbol-13-acetate/TPA for ≥10 d) [46]. The infected cells were fixed using a 10% formalin solution for 10 minutes at room temperature, i.e. for HCMV and MHV-68 at the time point of 4 d post-infection (d p.i.), HSV-1 24 h p.i., VZV 48 h p.i., and EBV-infected SVK-CR2 cells 4 d p.i. The SVK-CR2 cells were then permeabilized for indirect immunofluorescence (IF) staining of lamin A/C as described before [38, 42, 47], using rabbit mAb-lamin A/C as the primary antibody, incubated for 45 min at 37 °C, washed three times with PBS, and labelled with Alexa Fluor 555-conjugated goat anti-rabbit secondary antibody for 60 min at 37 °C. On-slide dephosphorylation was performed using lambda phosphatase (λ-PPase) to confirm phosphorylation of lamin A/C at pSer22. For this purpose, primary HFFs were infected with HCMV AD169-GFP (MOI 2), or remained uninfected. At 4 d pi., the cells were fixed, permeabilized, before dephosphorylation was performed using 1,600 units of λ-PPase under applied buffer conditions (i.e. λ-PPase in a buffer total volume of 500 µl, with 15 μl MnCl_2_, 466 μl DPBS, incl. 0.9 mM CaCl_2_, 0.5 mM MgCl_2_, also incl. 15 μl Protein MetalloPhosphatases/PMP; New England Biolabs, Ipswich, MA, USA). The slides were incubated for 3 h at 30 °C followed by blocking with Cohn II solution and primary antibody staining using rabbit pAb-pSer22 Lamin A/C and labelled with Alexa Fluor 647-conjugated goat anti-rabbit secondary antibody. Finally, cell samples were mounted with Vectashield Mounting Medium including DAPI for nuclear counterstaining (H-1700; Vector Laboratories, Burlingame, CA, USA). Confocal imaging was done using the TCS SP5 confocal laser-scanning microscope (Leica, Wetzlar, Germany). For the quantitative phenotypical evaluation of each sample, a total of 50 cells were counted in triplicates to determine the frequency of nuclear lamina deformation, and statistical analysis of data, given as mean values ± SD, was performed with Student’s t-test.

### Antiviral compounds

The antiviral compounds were dissolved in DMSO to prepare 10 mM-stock solutions, and aliquots were stored at -20 °C, i.e. the host-directed Pin1 inhibitor arsenic trioxide (As_2_O_3_; Sigma-Aldrich, Steinheim, Germany), the NEC-binding experimental drug P-020 (AiCuris AG, Wuppertal, Germany), and the developmental drug brincidofovir (BCV; Biomol GmbH, Hamburg, Germany)

### Quantitation of viral replication and antiviral activity of compounds

The antiviral activity of antiviral compounds against VZV Oka-GFP [25], HCMV AD169-GFP [9], and MHV-68-Luc [43] was performed according to the methodology described before.

## Results and discussion

### The nucleocytoplasmic egress of viral capsids is a mechanistically well-conserved regulatory process shared by *Herpesviridae* family members

**Fig. 1:**
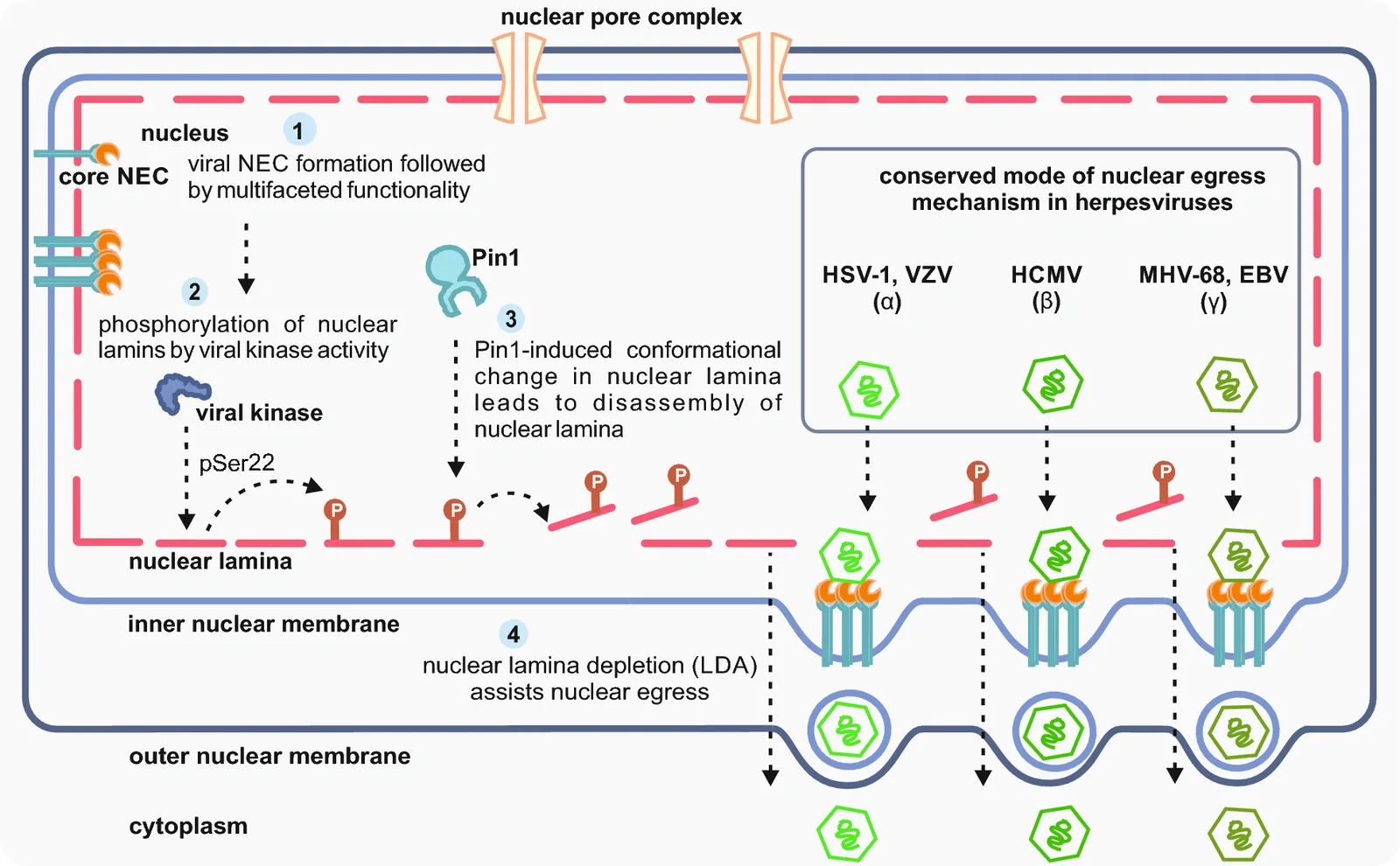
Proposed mechanism of nuclear egress conservancy in herpesviruses. During herpesvirus replication, the viral core nuclear egress complex (NEC), and its extension form of hexameric lattices, are formed at the inner nuclear membrane (INM) (step 1) [48]. The viral kinase specifically phosphorylates lamin A/C at Ser22 (step 2) [36]. The cellular *cis/trans* isomerase Pin1 further recognizes the pSer22-Pro23 motif of lamin A/C to induce conformational changes through *cis/trans* isomerization, ultimately disintegrating the nuclear lamina in the form of LDAs (step 3) [9, 36]. This depletion of the nuclear lamina provides access of herpesviral nuclear capsids to the INM and allows for the budding-mediated transition into the cytoplasm (step 4) [54]

Previous investigations of independent research groups, including our own studies, demonstrated the conserved principle of herpesviral nuclear egress [3, 5, 13, 14, 16–18, 20, 48–52]. Although the primary sequences of herpesviral core nuclear egress complexes are only conserved to a limited degree, the structures are highly preserved [19]. Interestingly, the regulatory events, based on the activity of NEC-associated cellular and viral proteins, await further clarification concerning their shared or unique mode of activity (Fig. 1). Our recent report about the role of viral protein kinase activity and the cellular prolyl *cis/trans* isomerase Pin1 in nuclear egress identified close parallels between human and animal cytomegaloviruses [9, 53].

### Serine22 phosphorylation of lamin A/C is consistently observed in herpesvirus infection

**Fig. 2:**
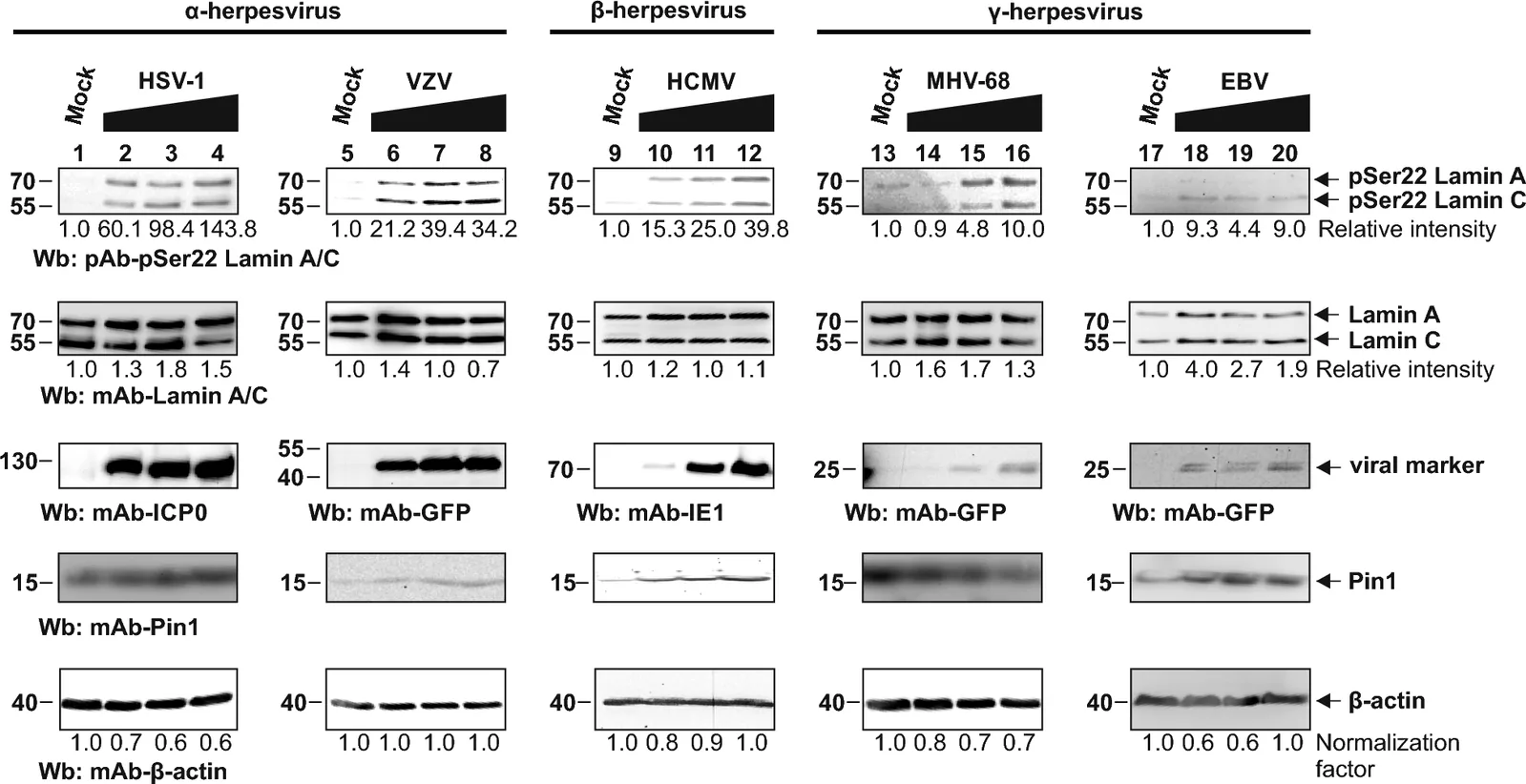
Serine22 phosphorylation of lamin A/C in herpesvirus-infected cells. HFFs were infected with selected herpesviruses, i.e. HSV-1 VP26-GFP, VZV Oka-GFP, HCMV AD169, MHV-68-GFP; SVK-CR2 were infected with EBV Akata-BX1-GFP. Infections were performed at increasing multiplicities of infection as indicated (MOIs approx. 0.1, 0.25, 0.5), except for HSV-1 and HCMV (MOI of 0.5, 1.0, 2.0), or remained uninfected. Cells were harvested at 4 d.p.i., with the exception of HSV-1 VP26-GFP at 24 h p.i. and VZV-GFP at 48 h p.i. Viral markers of infection were detected by Wb (ICP0 for HSV-1, IE1p72 for HCMV, GFP for VZV, GFP for MHV-68, GFP for EBV). Lamin A/C pSer22, lamin A/C total, and Pin1 were detected as indicated, and β-actin was used as an internal loading control. Individual band signal intensities were detected by densitometric analysis (AIDA Image Analyzer software v.4.23), were normalized to the β-actin signal, and further set into relative band intensity values as compared to the signals of mock-infected samples (Mock, lanes 1, 5, 9, 13, 17; mean signal values of three technical replicates are given)

In order to study the nuclear egress-related effect of herpesvirus infections on primary HFFs, cells were infected with selected α-, β-, and γ-herpesviruses, i.e. HSV-1, VZV, HCMV (previously also ChCMV and RhCMV [9]), MHV-68, and EBV, respectively. Here, a total of 3x10^5^ primary HFFs were seeded in a 6-well plate and infected with herpesviruses at increasing multiplicities of infection (MOIs approx. 0.1, 0.25, and 0.5), and for HSV-1 and HCMV (MOI of 0.5, 1.0, 2.0), while SVK-CR2 cells were used for EBV infection, or remained uninfected. The specific viruses used in this experiment were HSV-1 VP26-GFP, VZV Oka-GFP, HCMV AD169, MHV-68-GFP, and EBV Akata-BX1-GFP. Infected cells were harvested at 4 d p.i., except for HSV-1 at 24 h p.i., and VZV at 48 h p.i. The progress of infection was monitored by the Wb staining of viral marker proteins, i.e. ICP0 for HSV-1, IE1p72 for HCMV, GFP for VZV, GFP for MHV-68, and likewise GFP for EBV (Fig. 2, middle panels). The total signals for lamin A/C in HFFs (Fig. 2, second uppermost panels) remained at an almost constant level, whereby some slightly increased level was noted for infected compared to mock-infected cells (especially for VZV, HCMV, and EBV). The phosphorylated form of lamin A/C (pSer22) showed a clear upregulation in all herpesvirus-infected cells compared to the mock controls (Fig. 2, lanes 2-4, 6-8, 10-12, 14-16, and 18-20; for HSV-1, VZV, HCMV, MHV-68, and EBV, respectively). This upregulation of pSer22 appeared relevant, because it creates a binding motif for Pin1 in of lamin A/C, which may generally lead to Pin1-induced conformational changes and disassembly of the nuclear lamina. Interestingly, not in all cases a steady increase of the pSer22 signal was found within the range of increasing MOIs, possibly indicating a saturation level. It should be mentioned that the MHV-68 is a murine γ-herpesvirus (sometimes used as a model for human γ-herpesviruses like EBV), which principally can infect HFFs albeit a low permissiveness of productive infection. Interestingly, the pSer22-specific phosphorylation of lamin A/C could be detected (Fig. 2, lanes 14-16), also associated with nuclear lamina deformation (see below), using the cellular tools of this study (see below).

### Herpesvirus infection triggers LDA formation in recombinant lamin A/C-RFP HFFs

**Fig. 3:**
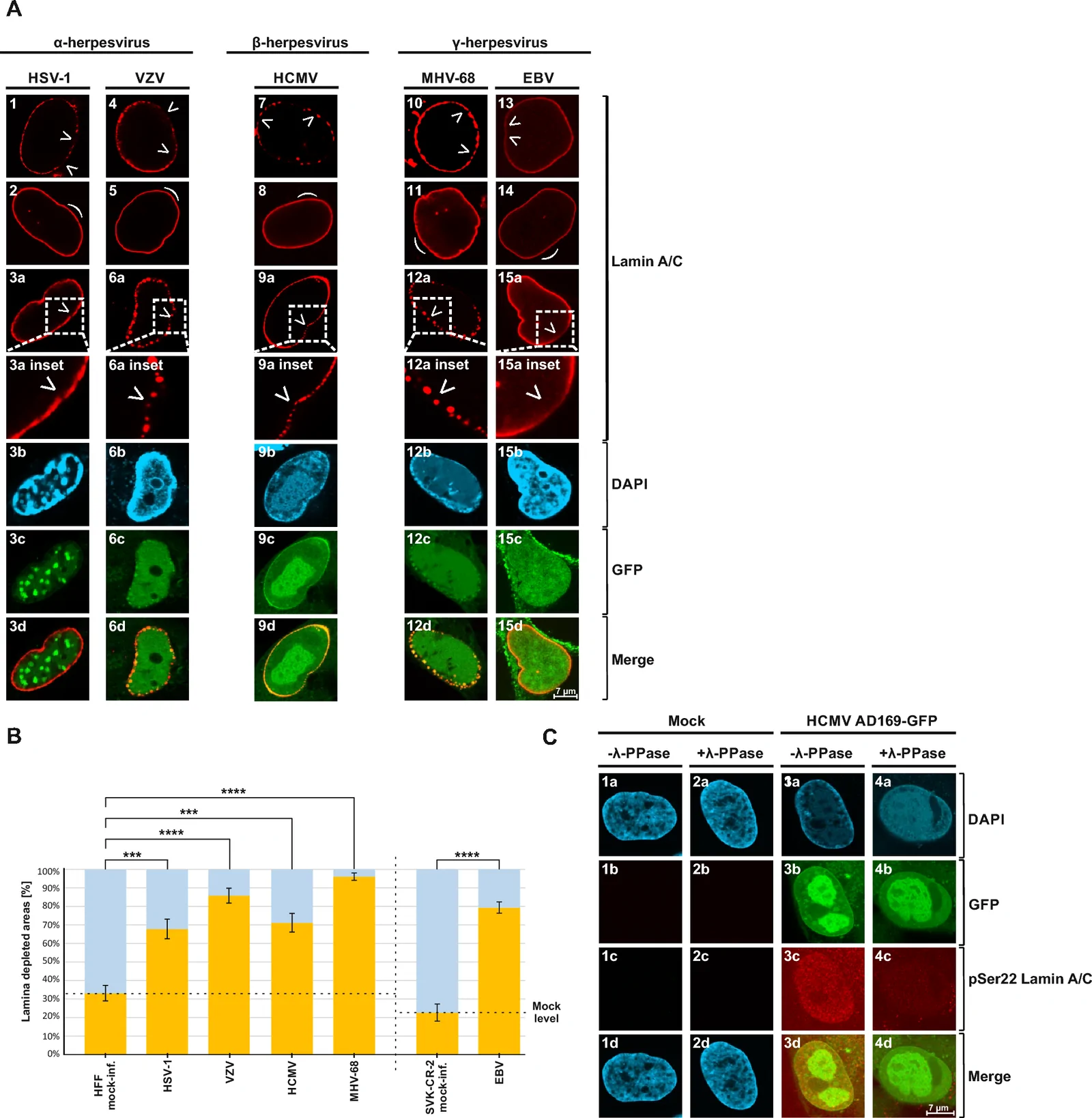
Conserved phenotype of LDA formation in herpesvirus-infected cells. (**A**) Viruses used were the α-herpesviruses HSV-1 VP26-GFP (MOI 1) and VZV Oka-GFP (MOI approx. 0.25), β-herpesvirus HCMV AD169-GFP (MOI 2), and γ-herpesviruses MHV-68-GFP (MOI approx. 0.25) and EBV Akata-BX1-GFP (MOI approx. 0.25). Dox-induced recombinant HFFs expressing lamin-A/C-RFP were used for infection (as well as SVK-CR2 cells infected with EBV-GFP in comparison). The infected cells were fixed with 10% formalin at the time point of 4 d p.i., except for HSV-1 at 24 h p.i. and VZV at 48 h p.i. SVK-CR2 cells were specifically immunostained to detect lamin A/C using rabbit mAb-Lamin A/C antibody. All cells were additionally subjected to a DAPI nuclear counterstaining. The phenotype of the nuclear lamina was observed using confocal microscopy, and the consistent nuclear rim was marked by white semicircles, whereas the lamina-depleted areas (LDAs) were marked by arrowheads. (**B**) The nuclear lamina phenotypes were quantified by counting entities of 50 cells in triplicates (i.e. virus-positive cells compared to mock control cells). The statistical analysis was performed using a Student’s t-test by comparing mock with infected cells, ***, P≤0.001; ****, P≤0.0001. (**C**) HFFs were infected with HCMV AD169-GFP (MOI 2) or remained uninfected and fixed at 4 d p.i. The cells were treated with (+) or without (-) lambda phosphatase (λ-PPase). Phosphorylation-specific immunostaining of the nuclear lamina was performed using pAb-pSer22 Lamin A/C. For conditions of λ-PPase treatment, see Methods section

The effect of herpesvirus-induced LDA formation was analyzed using confocal microscopy. The dox-induced recombinant lamin A/C-RFP HFFs (and in comparison, EBV-permissive SVK-CR2 epithelial cells) were infected with herpesviruses as described in the methods section. Upon infection, the LDA phenotypes were consistently observed across representative herpesvirus species in infected cells. The analyzed α-, β-, and γ-herpesviruses expressed comparable phenotypes of LDA formation, i.e. HSV-1 VP26-GFP (Fig. 3A, as indicated by arrowheads, images 1, 3a-d), VZV Oka-GFP (images 4, 6a-d), HCMV AD169-GFP (images 7, 9a-d), MHV-68-GFP (images 10, 12a-d), and EBV (images 13, 15a-d). The quantitation indicated a substantial increase in the frequency of LDA formation through virus infection, compared to uninfected controls (see HFF mock-inf. and SVK-CR2 mock-inf.), ranging from 68.0% to 96.0% (Fig. 3B). All virus-specific LDA effects showed statistical significance (P≤0.001 or P≤0.0001). This finding suggests the conservancy in the exhibition of herpesvirus-induced lamin A/C deformation during the course of the infection. As an additional control, we included an immunostaining of pSer22 phosphorylation of lamin A/C (Fig. 3C). The phospho-specific signal was massively induced by HCMV infection (Fig. 3C, image 3c) compared to mock-infected samples (Fig. 3C, image 1c), thus supporting the indication that lamin phosphorylation drives the LDA phenotype in this reporter system. In addition, lambda phosphatase (λ-PPase) treatment reversed this intranuclear staining signal (Fig. 3C, image 4c), thereby confirming our assay specificity. Notably, in our earlier study, we have also used lamin A/C-RFP mutants, such as a phospho-mimetic (S22E) and a phosphoserine-deficient version (S22A). Those data have further illustrated that phosphorylation of lamin A/C on Ser22 is linked to the LDA phenotype. The S22A mutant showed significantly reduced levels of cytomegalovirus-induced LDA formation, whereas the S22E mutant produced an intermediate state of LDA phenotype, which was partly independent of viral infection [9, 36]. This finding underlined the link between phosphorylation of lamin A/C on Ser22 and viral phenotypes of LDA formation.

### HCMV viral kinase pUL97 triggers the LDA formation to promote nuclear egress

**Fig. 4:**
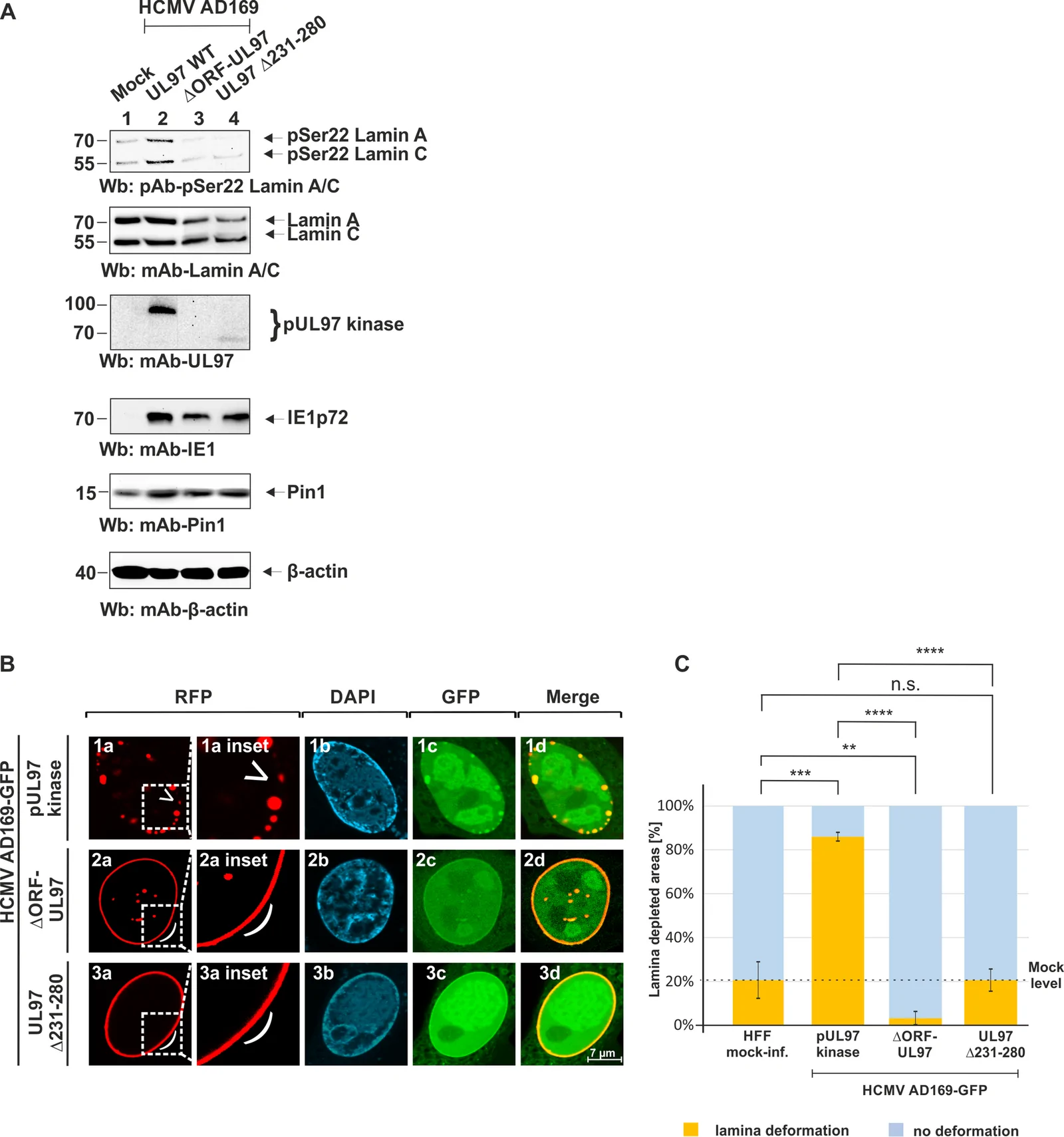
The functional role of human cytomegalovirus pUL97 kinase in nuclear egress. (**A**) The dox-induced recombinant lamin A/C-RFP HFFs were infected with HCMV AD169-GFP wild-type (WT, pUL97 kinase unaffected), the mutants ΔORF-UL97 and UL97 Δ231-280, or remained uninfected. The Wb analysis was performed by using the indicated antibodies. Note, the differences in expression patterns and phosphorylation signal levels detected for the different types of infection. (**B**) For microscopic analysis of IF staining, the cells were fixed at 4 d p.i. and counterstained with DAPI. The LDA phenotype was analyzed by confocal imaging, in that nuclear lamina/LDA samples were qualitatively assessed by comparing structural rearrangements between the infection settings. A consistent nuclear lamina rim pattern was marked by white semicircles, while the lamina-depleted areas (LDAs) were marked by arrowheads. (**C**) The quantitative assessment was achieved by counting 50 cells in triplicates (i.e. virus-positive cells compared to mock control cells). The statistical analysis was performed using a Student’s t-test, *, P≤0.05; **, P≤0.01; ***, P≤0.001; ****, P≤0.0001

To study the role of viral kinase activity in LDA formation during nuclear egress, we used a viral kinase deletion mutant of HCMV, AD169-GFP ΔORF-UL97 [27], and a cyclin-binding deficient mutant of HCMV, AD169-GFP UL97 Δ231-280 [55]. The mutant ΔORF-UL97 completely lacks viral kinase activity, due to a deletion of the entire open reading frame (ORF) UL97 from the viral genome, while UL97Δ231-280 carries a deletion in the interface 2 (IF2) of its cyclin binding domain, which is required for attaining full kinase activity [55]. For addressing the impact on site-specific lamin A/C phosphorylation (pSer22), HFFs were infected with either of these two HCMV kinase mutants and compared to the parental wild-type (WT). Due to the fact that the HCMV mutants expressed a delayed replication kinetics [55], infected-cell supernatants of ΔORF-UL97 and UL97 Δ231-280 were first prepared by pre-culture infection, to harvest these infectious supernatants as virus stocks at late time points (> 1 week, when virus-induced cytopathic effects became dominant in approx. 80% of cells). These freshly prepared virus inocula were then used to achieve comparable MOIs with all three viruses (MOI approx. 1; Fig. 4) in dox-induced recombinant lamin A/C-RFP HFFs. At 4 d.p.i., cells were harvested and total lysates were subjected to Wb analysis (Fig. 4A). Viral immediate early protein expression (IE1p72) and kinase pUL97 were stained as specific infection markers (middle panels; note deletion of pUL97 in lane 3, and truncation of pUL97 in lane 4). Pin1 upregulation was noted as a general consequence of infection (Fig. 4A, second lowest panel, lanes 2-4, compared to mock-infected control in lane 1), and the total lamin A/C staining was consistently detectable (second uppermost panel, lanes 1-4). Of note, the phosphorylated variety pSer22 of lamin A/C, showed clear differences in quantitative terms (upper panel). While WT infection (lane 2) produced the expected pSer22 upregulation over the mock-infected control (lane 1), the levels found for the two HCMV kinase mutants, ΔORF-UL97 and UL97 Δ231-280, were substantially lower, (Fig 4, lanes 3-4). Thus, due to the absence of kinase activity (ΔORF-UL97) or due to the lack of kinase-stimulatory pUL97–cyclin interaction (UL97 Δ231-280 [55]), respectively, a significant loss-of-function in the upregulation of lamin A/C pSer22 was identified. This strongly confirmed our postulate that pUL97 triggers the generation of the pSer22-specific Pin1 binding motif, which consequently promotes the formation of LDAs during HCMV infection. The latter activity was then determined by microscopic evaluation of LDA formation (Fig. 4B-C). The comparative analysis of the HCMV mutants and WT revealed that the mutants ΔORF-UL97 and UL97 Δ231-280 displayed an LDA-negative phenotype, as characterized by a nuclear lamina structure indistinguishable from uninfected cells (Fig. 4B, images 2a-d and 3a-d). The quantitative evaluation confirmed the significant difference between WT pUL97 kinase activity and the two mutants (Fig. 4C). Interestingly, the mutant levels were comparable to, or even lower than, mock-infected control levels, which might suggest a mutant viral interference with the LDA basal level seen in mock-infected cells, which might represent cellular endogenous LDA-like effects. Overall, the findings strongly support the role of HCMV kinase activity in the site-specific lamin A/C phosphorylation and LDA formation.

### Pin1 and NEC inhibitors restore normal phenotype of the nuclear lamin in herpesvirus-infected cells

**Fig. 5:**
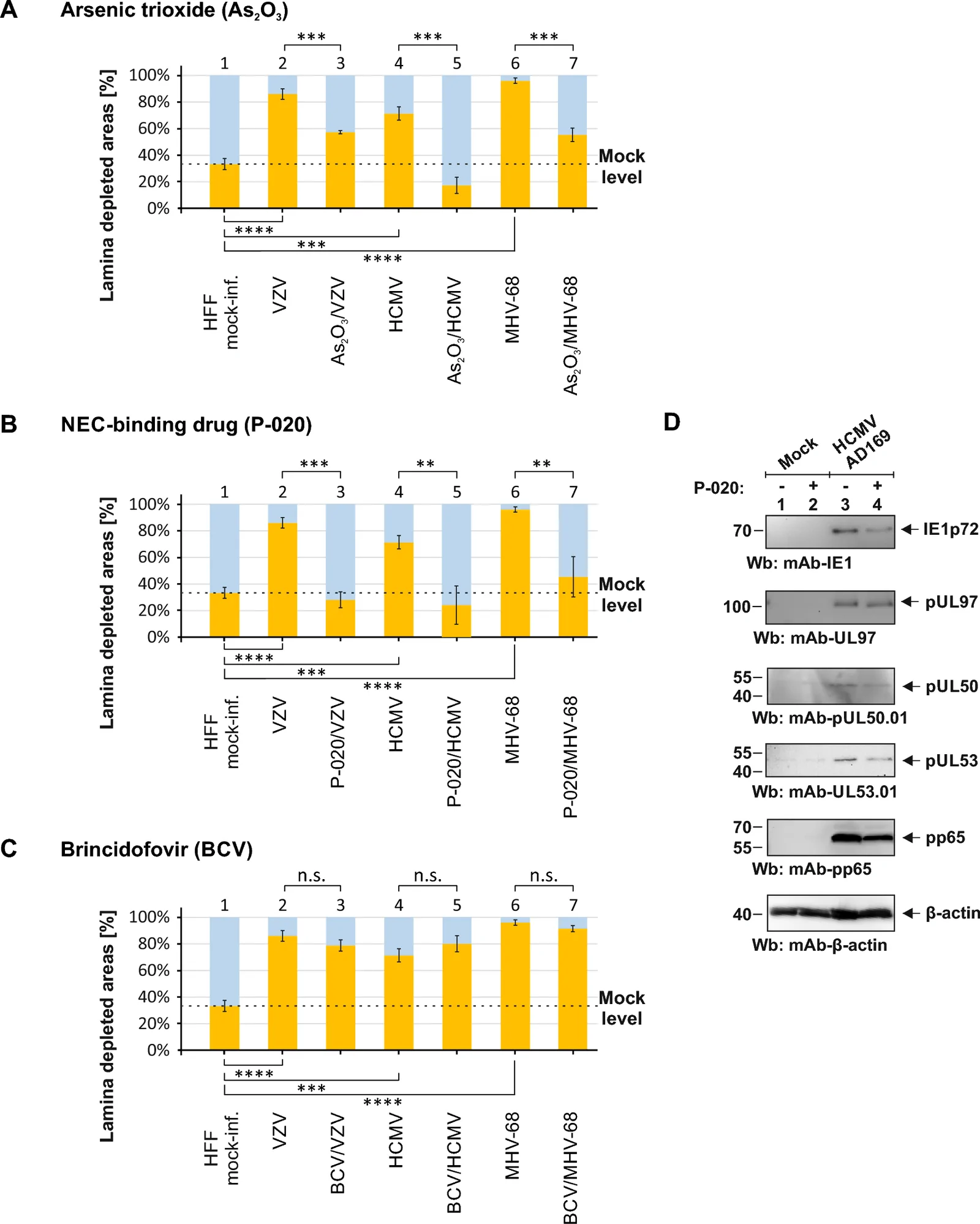
Quantitation of LDA formation in herpesvirus-infected reporter cells under the influence of cellular Pin1 and viral NEC inhibitors. Dox-induced recombinant lamin-A/C-RFP expressing HFFs were infected with VZV-GFP (MOI approx. 0.25), HCMV AD169-GFP (MOI 2), or MHV-68-GFP (MOI approx. 0.25). At 4 h p.i., cells were treated with antiviral compounds, i.e. in (**A**) the host directed Pin1 inhibitor As_2_O_3_ (2.2 µM), (**B**) viral NEC-directed inhibitor P-020 (20 µM), or (**C**) the developmental antiherpesviral drug BCV (0.01 µM). The rationale for using these distinct compound doses was based on the previous antiviral profiling of each compound for the respective viruses, i.e. each representing the double dose of the half-maximal effective antiviral concentration (2x EC_50_), since this has proven to be successful before [9]. The antiviral compounds were refreshed daily (with the exception of low-level replication of MHV-68-GFP, maintained under continuous unrefreshed drug treatment). At 4 d p.i., cells were fixed (except VZV Oka-GFP at 48 h p.i.). The nuclear lamina phenotypes were quantified by counting 50 cells in triplicates (i.e. virus-positive cells compared to mock control cells). The statistical analysis was performed using Student’s t-test, **, P≤0.01; ***, P≤0.001; ****, P≤0.0001; n.s., not significant, P>0.05. (**D**) Additional Wb analysis was performed to demonstrate that the NEC inhibitor P-020 does not affect the expression levels of the viral kinase, NEC components, or further viral proteins. HFFs were infected with HCMV AD169 (MOI 2), treated with P-020 (20 µM) or left untreated, and used for the preparation of total cellular lysates at 4 d p.i., followed by SDS-PAGE/Wb immunostaining with the indicated antibodies

Previously, we reported that the application of viral pUL97 kinase inhibitor maribavir (MBV) or a host-directed Pin1 inhibitor (As_2_O_3_) exerted antiviral activity, and restored the frequency of LDA formation to a mock-infected cell level, when comparing primate CMVs [9]. Here, we evaluated this phenotype of LDA formation, under the treatment with Pin1 inhibitor As_2_O_3_, NEC inhibitor P-020, or the developmental antiherpesviral drug brincidofovir (BCV), for additional herpesviruses of the three subfamilies. The chosen compounds have been evaluated in different contexts by our earlier studies, i.e. BCV against VZV Oka-GFP [48], As_2_O_3_ against HCMV [9], and P-020 against VZV and HCMV [28]. Especially, the pharmacophore-based identification of the antiviral nuclear egress complex (NEC) inhibitor 020 (P-020) is currently under investigation by our group and other researchers [48]. The compound was selected through a computational approach (pharmacophore modelling), utilizing the available crystal structures of herpesviral core NECs, and was shown to inhibit nuclear egress and exhibit broad antiherpesviral activity [28]. Further investigations will be required to provide deeper insight into the mechanistic details of P-020. BCV has been chosen as a potentially NEC-independent reference inhibitor. Its antiviral mode-of-action (MoA) has originally been based on the known mechanism of the parental drug, cidofovir (CDV), acting as an inhibitor of herpesviral DNA polymerase, with a relatively broad spectrum of activity [56]. In addition, stimulated by the finding that BCV exerts antiviral activity even against non-herpesviruses (such as monkeypox, adeno-, and polyomaviruses), the understanding of its underlying MoA suggests a more complex situation [57], which may include the targeting of additional viral and/or cellular polymerases. In our present study, the conditions of virus infection, antiviral drug treatment, and the readout systems have been described elsewhere [9, 25, 28, 43], and novel data have been set into the comparison with previous findings (Table 1). Interestingly, these three antivirals all exert some degree of broad-spectrum antiherpesviral potency (including VZV, HCMV, and MHV-68), with detectable EC_50_ values in the range of nanomolar (BCV), low micromolar (As_2_O_3_), or intermediate micromolar concentrations (P-020) (Table 1). Consequently, we applied these antiviral treatments in our reporter model with dox-induced recombinant lamin A/C-RFP HFFs. To this end, cells were infected with VZV Oka-GFP, HCMV AD169-GFP, or MHV-68-GFP, and inhibitor treatment was performed for the assessment of LDA effects (Fig. 5; As_2_O_3_ administered at 2.2 µM, P-020 at 20 µM, and BCV at 0.01 µM; all with daily culture media/compound refreshment). As a relevant finding, under the As_2_O_3_ treatment (Fig. 5A) or P-020 treatment (Fig. 5B), the frequency of LDAs reduced significantly in compound-treated/infected versus untreated/infected cells (Fig. 5A, compare columns 2/3, 4/5, and 6/7). In contrast, BCV treatment did not show a comparable reduction in the virus-induced LDA frequency (Fig. 5C). In order to rule out that the NEC inhibitor P-020 might affect the expression levels of the viral kinase or NEC components, an additional Wb analysis was performed (Fig. 5D). To this end, HFFs were infected with HCMV AD169 (MOI 2) and subjected to the treatment with P-020 compared to untreated samples. The Wb immunostaining of samples harvested at 4 d p.i., using the indicated antibodies, demonstrated that a very low P-020-mediated impact on the expression of viral proteins was detectable. This was indicated by mostly unaltered levels of immediate early protein (IE1p72), early proteins (pUL97, pUL50, and pUL53), or early-late (pp65) proteins (Fig. 5D, lanes 3-4). Combined, these results strongly argued for a specific impact of the cellular Pin1 inhibitor (As_2_O_3_) and the viral NEC inhibitor (P-020) in regard of LDA formation and viral nuclear egress efficiency.

**Table 1:** Summary of half-maximal effective antiviral concentrations, given as EC_50_ values (compared to cytotoxic concentration values/CC_50_, and selectivity indices/SI) of [mechanistically distinct] antiherpesviral drugs

**Compound**	**α-herpesvirus** **VZV**	**β-herpesvirus** **HCMV**	**ɣ-herpesvirus** **MHV-68**
BCV [viral DNA polymerase inhibitor]	EC_50_ 0.065 ± 0.035 µM [25] (CC_50_ 5.9 µM, SI 91)	0.00015 ± 0.00006 µM (CC_50_ 5.9 µM, SI 40000)	0.01 ± 0.003 µM [43] (CC_50_ >10.0 µM, SI >1000.0)
As_2_O_3_ [Pin1 inhibitor]	EC_50_ 3.5 ± 1.5 µM [9] (CC_50_ 27.0± 4.0 µM, SI 7.7)	1.1 ± 0.4 µM [9] (CC_50_ 27.0 ± 4.0 µM, SI 24.5)	7.3 ± 2.7 µM (n.d.)
P-020 [NEC inhibitor]	EC_50_ 10.3 ± 1.7 µM [28] (CC_50_ 33.90 ± 2.5 µM, SI 3.3)	6.8 ± 4.6 µM [28] (CC_50_ 33.90 ± 2.5 µM, SI 5.0)	33.0 ± 11.0 µM (n.d.)

The antiviral activity (mean EC_50_ values ± SD) of the inhibitors against HCMV AD169-GFP and VZV Oka-GFP was determined using the GFP-based virus replication assay in primary HFFs as described before [9]; in case of MHV-68-Luc, the firefly luciferase (Luc) reporter assay was performed in Vero cells [43]. Levels of cell viability were accessed by the Neutral Red uptake assay (NRA), measured at time points identical to those applied for virus infection, as described before [9, 43]; n.d., not determined.

## Conclusion

As exemplified in this study and by previous reports, the replication efficiency of herpesviruses is dependent on multifold nuclear virus–host interactions. Hereby, the optimal fine-regulation of nucleocytoplasmic capsid egress represents a rate-limiting step. The quantitative assessment of herpesviral nuclear egress activity has been a longstanding goal of several research approaches, including the phenotypic characterization of viral NEC mutants as well as the measurement of antiviral potency of NEC inhibitors [14, 58–60]. Our own methodological experience with the assessment of viral egress spans a variety of readout systems. These include (i) virus-specific quantitative PCR detection of genomic DNA based on infected-cell fragmentation (qPCR-NEAssay), (ii) electron microscopic (EM) capsid analysis, (iii) confocal staining of viral NEC nuclear rim formation (NRFAssay), (iv) infection kinetics of HCMV NEC mutants in NEC-complementing cells (NEC complementation assay), (v) NanoBiT®-based NEC interaction assay (NanoBiT-NEC assay; Promega, Walldorf, Germany), (vi) detection of viral cytoplasmic assembly compartments (cVAC assay), (vii) *in vitro* assembly-based NEC/viral protein coimmunoprecipitation (NEC-CoIP) [9, 14, 25, 61–65], and, as reported in this study, (viii) the quantitative assessment of herpesviral LDA formation (LDA assay). The advantage of cell-based assays, including microscopic detection systems (i.e. EM, NRFA, cVAC, and LDA assays), is seen in the direct visualization of intracellular egress-specific effects, while a disadvantage may arise from the technically demanding procedures. In comparison, the advantage of cell-based assays, including very sensitive quantitative systems (qPCR-NEA, NanoBiT-NEC, in part also NEC complementation, and NEC-CoIP assays), has been experienced in their adaptability towards a medium-scale screening of increasing sample numbers, whereas a disadvantage can sometimes be given by a certain inherent assay variability. For these reasons, the choice of a most appropriate nuclear egress measurement should be made dependent on the individual research question and assay conditions.

Several investigations described the formation of nuclear lamina-depleted areas (LDAs) as a phosphorylation-triggered local rearrangement of the rigid lamina meshwork within the nuclear envelope, thereby facilitating herpesviral capsid egress. Our current work suggests that the site-specific phosphorylation pSer22 of lamin A/C and LDA formation is a phenomenon consistently observed during the infection with various herpesviruses. In the case of HCMV, viral kinase pUL97 is mainly responsible for pSer22 phosphorylation of lamin A/C, thereby playing the central role in LDA formation. It should be mentioned that although viral kinases mediate the lamin A/C phosphorylation, successful nuclear egress may also require host cellular kinases. This accessory role of cellular kinases has been demonstrated for HCMV [6, 62, 66] and HSV-1 [67, 68]. The regulatory importance of these kinases, such as protein kinase C (PKC) isoforms, cyclin-dependent kinases 1 and 2 (CDK1/2), and others, for lamin A/C phosphorylation in herpesvirus-infected cells and nuclear egress efficiency, however, awaits further clarification [69, 70]. Moreover, we like to emphasize that lamin A/C phosphorylation can be utilized as a quantifiable parameter of herpesviral nuclear egress activity, but that both the lamin A/C p Ser22 marker and the LDA phenotype are only a part of the entire nuclear egress mechanism. An important hallmark is, that in response to Ser22 phosphorylation, Pin1 can be recruited to its phospho-specific minimal binding motif in lamin A/C, pSer22-Pro23. This site-specific binding has been assigned to the consequence of Pin1-mediated lamin *cis/trans* isomerization and the shaping of LDAs [7–9, 48].

With the novel findings of the present study, we postulate that these regulatory events are not specific for HCMV and related β-herpesviruses, but likewise play a similar role in the replication of herpesviruses belonging to other subfamilies. Our autofluorescent reporter cell model enabled the quantitation of viral LDA formation in the context of variable conditions of infection. These included virus species, host cell types, time points of infection, viral expression patterns, and effects of inhibitor treatment. In particular, the study focused on inhibitory small molecules exerting antiviral activity on the basis of cellular Pin1 or viral NEC inhibition. Both targeting options are functionally linked to viral nuclear egress regulation, thus significantly reducing the frequency of LDA formation in several analyzed examples of herpesvirus infection. Combined, the study illustrates the broad applicability of this cell-based reporter system, also demonstrating consistencies in the nuclear egress regulation among herpesviruses, and thereby supporting our translational approach to utilize egress-associated proteins as novel antiviral drug targets.

## Data Availability

All datasets generated were presented in the current study.
